# Systematic Review of Protein Signatures for Clinical Monitoring of Osteonecrosis of the Jaw: Meta-Analysis and Insights from Bioinformatics-Driven Proteomics

**DOI:** 10.3390/proteomes14020029

**Published:** 2026-06-10

**Authors:** Helena Oliveira Deróbio, Isabela dos Reis Souza, François Isnaldo Dias Caldeira, Fernanda Gonçalves Basso, Taisa Nogueira Pansani

**Affiliations:** 1 Department of Dental Materials and Prosthodontics, School of Dentistry, São Paulo State University (UNESP), Humaitá Street, 1680, Araraquara 14801-903, SP, Brazil; helena.derobio@unesp.br (H.O.D.); isabela.r.souza@unesp.br (I.d.R.S.); 2 Department of Morphology and Pediatric Dentistry, School of Dentistry, São Paulo State University (UNESP), Humaitá Street, 1680, Araraquara 14801-903, SP, Brazil; francois.isnaldo@unesp.br; 3 Department of Diagnosis and Surgery, School of Dentistry, São Paulo State University (UNESP), Humaitá Street, 1680, Araraquara 14801-903, SP, Brazil; 4 Department of Physiology and Pathology, School of Dentistry, São Paulo State University (UNESP), Humaitá Street, 1680, Araraquara 14801-903, SP, Brazil; f.basso@unesp.br

**Keywords:** osteonecrosis, bisphosphonates, proteome

## Abstract

Background: Several studies have investigated the clinical and immunological aspects of medication-related osteonecrosis of the jaw (MRONJ). However, the underlying immunological mechanisms and signaling pathways involved in its pathophysiology remain incompletely understood. This systematic review and meta-analysis, complemented by bioinformatics analyses, aimed to identify proteomic biomarkers associated with MRONJ. Methods: Six databases (PubMed, Embase, Scopus, Web of Science, Cochrane Library, and VHL) were searched, along with gray literature and manual searches. Observational studies in English comparing proteomic profiles of individuals with and without MRONJ were included. Study selection and data management were conducted using EndNote™ X8 and Rayyan.ai, and risk of bias was assessed using the QUADOMICS tool. Functional enrichment analysis was performed using g:Profiler and Reactome, and interaction networks were constructed using GeneMANIA, STRING, and MetaboAnalyst (Cytoscape program; version 3.10.1). Meta-analysis was performed in RStudio (R-4.5, Rstudio extension 2025.05.1+513) (α = 0.05). Results: Three studies were included in the review, and two in the meta-analysis. The meta-analysis showed higher salivary levels of Apolipoprotein B-100 (APOB), Apolipoprotein A-II (APOA2), and Heparin Cofactor 2 (SERPIND1) in MRONJ patients, while the protein Keratin (KRT16) showed reduced levels without statistical significance. Bioinformatics analyses indicated involvement in lipid metabolism, impaired tissue repair, and inflammatory and immune responses. Conclusions: These findings suggest altered salivary proteomic signatures in MRONJ for APOB, APOA2, SERPIND1, and KRT16 proteins.

## 1. Introduction

Medication-related osteonecrosis of the jaw (MRONJ) is defined as a debilitating condition first reported by Marx in 2003 [1], characterized by the presence of exposed bone in the maxillofacial region for a minimum period of eight weeks in patients currently or previously treated with antiresorptive or antiangiogenic drugs and with no history of radiotherapy in the head and neck region [2]. The presence of this lesion compromises the patient’s quality of life, posing challenges to dental treatments and essential activities such as chewing and social interaction [3,4].

Antiresorptive agents are widely used in the management of diseases that result in unbalanced bone formation and resorption, such as osteoporosis and various malignant neoplasms [5]. Among these drugs, bisphosphonates (BPs) [6] stand out, a fundamental subclass characterized by a molecular structure containing two phosphonate groups linked to a non-hydrolyzable carbon. This chemical configuration gives BPs a high affinity for mineralized tissue through calcium ion chelation [7]. Consequently, this affinity promotes selective accumulation of the drug in areas of intense bone remodeling, which explains its preferential concentration in the mandible and maxilla due to the high turnover rate in these regions [8]. Although therapeutically effective, the prolonged accumulation and local action of BPs are associated with decreased angiogenesis and impaired bone repair, factors that, combined with increased inflammation, predispose the patient to the development of osteonecrotic areas [8].

Treatment for MRONJ is determined according to the staging of the disease and can range from the use of pain medication and antibiotics, as well as antimicrobial mouthwashes, laser treatments, and even surgical treatment for debridement of the lesion [9]. The resolution of MRONJ is recognized as difficult and uncertain, requiring a multidisciplinary approach that prioritizes prevention of the condition and rigorous patient education [10]. Currently, the diagnosis of MRONJ is based primarily on clinical evaluation and imaging tests, such as X-rays and computerized tomography scans, which detect already established structural changes [11,12]. However, these methods are insufficient for the early identification of individuals at risk of developing bone necrosis before the onset of clinical signs [13]. Given this limitation, the search for biomarkers for the predictive and early diagnosis of MRONJ is crucial to optimize the management and prognosis of this condition.

To meet the demand for molecular diagnostics, proteomic analysis has established itself as a powerful tool in the search for biomarkers in various body fluids and tissues, such as blood, urine, or saliva [14]. This method of analysis (proteomics) allows the quantification of hundreds of proteins at once, providing high-dimensional data that is essential for understanding pathological processes and detecting physiological changes before clinical signs manifest themselves [15,16].

The usefulness of proteomic analysis in identifying biomarkers has been widely demonstrated in complex inflammatory and metabolic pathologies that, like MRONJ, involve critical interactions between oral tissues and systemic conditions [17]. Studies using mass spectrometry in patients with metabolic imbalances and periodontitis, for example, have identified proteins such as Titin (TTN) and Enolase-1 (ENO-1) as promising biomarker candidates, capable of reflecting the onset of tissue degradation even before the manifestation of clinical signs [18]. Although the clinical application of these specific proteins is still in the validation phase, these findings demonstrate the ability of proteomics to detect “molecular signatures” that conventional imaging tests cannot capture [18]. Therefore, applying this same technology to the context of MRONJ represents a strategic advance in overcoming the dependence on late diagnoses.

However, despite the potential of this tool in different biological samples, there is a gap in the literature regarding the synthesis of molecular data specific to MRONJ. To date, this is the first systematic review with meta-analysis investigating the proteome in the context of bisphosphonate-induced osteonecrosis of the jaw, comparing the findings with groups of patients who did not have this condition and healthy controls, with the aim of identifying diagnostic proteins and understanding the pathophysiological mechanisms involved in this process.

## 2. Materials and Methods

### 2.1. Study Protocol and Registration

This systematic review with meta-analysis and proteomics insights followed the guidelines proposed by the PRISMA (Preferred Reporting Items for Systematic Reviews and Meta-Analyses) checklist [19]. The protocol for this study was registered on the PROSPERO platform under registration number (CRD420251236748).

### 2.2. PECOS Question

The research question formulated was: “In proteomic studies, are there differences in abundant proteins when comparing individuals without MRONJ with individuals with MRONJ?” For the research question, we used the acronym PECOS:

- Population (P): Individuals undergoing proteomic analysis in biological samples for MRONJ (individuals exposed or not exposed to MRONJ);
- Exposure (E): Patients over 18 years of age diagnosed with MRONJ according to the classification proposed by the American Association of Oral and Maxillofacial Surgeons (AAOMS) were to be included;
- Comparison (C): Clinically healthy individuals or controls for osteonecrosis of the jaws;
- Outcomes (O): Proteomics techniques employed to identify novel biomarkers associated with MRONJ;
- Study design (S): Only nonrandomized study types were to be included.

### 2.3. Eligibility Criteria

We included in this systematic review original observational studies in humans (cross-sectional, case–control, and cohort) comparing individuals with MRONJ and without MRONJ in terms of the average protein differentially abundant proteins profile of the sample and/or concentration obtained using proteomic methods. Articles in English, available for reading. We excluded from this systematic review studies in other languages, patients undergoing treatment for MRONJ using drugs other than bisphosphonates, reviews, letters to the editor, conference publications, studies in cells or animals, and studies not available for reading.

### 2.4. Literature Search Strategy

On 1 August 2025, an electronic search was conducted using the PubMed databases via MEDLINE, EMBASE, Elsevier’s Scopus, the Virtual Health Library (VHL), Clarivate Analytics’ Web of Science, and the Cochrane Library, with no restrictions on publication date. Relevant articles will be identified through database searches using the following terms: “bisphosphonate-associated osteonecrosis of the jaw” AND “proteomics” with the respective search acronyms, which can be seen in Supplementary Material. In addition, systematic searches were conducted in gray literature, such as Google Scholar, to identify potential studies that could be included for analysis in this systematic review with meta-analyses and proteomic insights. Manual searches were also conducted in the main relevant journals on the subject, such as: with Proteomics and Systems Biology, Proteomes, Clinical Proteomics, Journal of Proteomics, Journal of Proteome Research, Clinical Oral Investigations, The Journal of Mass Spectrometry, International Journal of Molecular Sciences, Oral Diseases, British Journal of Oral and Maxillofacial Surgery, Journal of Dental Research, Oral Surgery, Oral Medicine, Oral Pathology and Oral Radiology, and Journal of Oral and Maxillofacial Surgery.

### 2.5. Reviewer Training

Before conducting this systematic review on the search for new proteomic biomarkers for the clinical monitoring of osteonecrosis of the jaw, two evaluators (I.R.S. and H.O.D.) underwent training and calibration to ensure greater accuracy and reproducibility in the analysis process. The level of agreement between the evaluators was measured using Cohen’s Kappa coefficient test, which showed a satisfactory value of 0.908 for reading titles and abstracts (screening stage-Prisma Flowchart) and 0.976 for complete reading (included study stage-Prisma Flowchart). The analyses were performed using the statistical program IBM SPSS Statistics 24 for Windows (Version 24.0. IBM Corp., Armonk, NY, USA).

### 2.6. Study Selection Strategy and Extraction

The process of analyzing, selecting, and including studies was carried out in three important stages. Initially, an independent researcher (F.I.D.C.) conducted strategic searches in the selected databases and then exported all articles found to EndNoteTM X8 (Thomson Reuters, New York, NY, USA), with the main objective of excluding duplicates. Next, two blinded evaluators (I.R.S. and H.O.D.) individually excluded studies based on titles and abstracts, using the inclusion and exclusion criteria established for this study. After completing this important step, the evaluators (I.R.S and H.O.D.) read the studies in full and compiled the information needed to compose the systematic review with meta-analyses and insights into proteomics. In case of disagreement between the evaluators, a third expert was asked to make the final decision on the inclusion of the study (T.N.P.). Rayyan: AI-Powered Systematic Review Management Platform (https://www.rayyan.ai/), accessed on 10 October 2025, was used for the synthesis and selection of articles [20].

The information was extracted from each study and compiled independently in Microsoft Excel. The variables collected in this study were: Author/Year; Study design; Sample medium; Sample size; Demographic profile (Case/Control); Diagnostic criteria (Case/Control); Analysis method (model); Number of proteins identified, and Bioinformatics approach. After summarizing the information for the qualitative syntheses, the third evaluator (T.N.P.) carefully checked each piece of information extracted by the two reviewers.

### 2.7. Risk of Bias in Individual Studies

Two authors (I.R.S. and H.O.D.) individually assessed the risk of bias in the included studies. This assessment was agreed upon using a modified version of the NIH Quality Assessment Tool for Observational Cohort and Cross-Sectional Studies and the QUADOMICS tool, developed specifically to assess omics [21,22]. Discrepancies found were analyzed and resolved through a consensus workshop with two systematic review experts (T.N.P. and F.I.D.C.).

### 2.8. Identification of Differences in Abundant Proteins in Proteomic Studies

The set of all differential proteins with increased/decreased abundance was obtained from the studies included in this review based on the type of biofluid and the target population analyzed. To achieve greater accuracy in identifying the signatures obtained, all identification codes “Entry Name” were reanalyzed in UniProtKB [23,24] to standardize names, isoforms, abbreviations, and coding genes. Proteins deleted by UniProtKB were excluded. Only differentially abundant proteins with statistically significant values (*p* < 0.05) were included for bioinformatic analysis and meta-analysis.

Information regarding the proteomic platform, protein quantification approach, normalization procedures, and handling of values was extracted from the included studies whenever available. The reported quantification strategies included label-free quantification, iTRAQ, and other mass spectrometry-based methods. Since the present review relied exclusively on published data, missing values were not imputed. Proteins were included in the meta-analysis only when sufficient quantitative information was available; otherwise, they were considered for qualitative synthesis and complementary bioinformatics analyses.

### 2.9. Bioinformatic Approach

#### 2.9.1. Venn Analysis

Proteins with increased/decreased abundance identified in each study were subjected to a comparative analysis using the Venny 2.1 tool available on the platform (https://bioinfogp.cnb.csic.es/tools/venny/index2.0.2.html). The information was consulted on 23 December 2025.

#### 2.9.2. Gene Networking Analysis

To predict the effects of bisphosphonate on the jaw, a gene-gene interaction network was constructed using the codes of the genes responsible for transcribing the proteins identified in the proteomic signature. For this purpose, we used the Cytoscape program (version 3.10.1) with the GeneMANIA extension [25,26]. GeneMANIA constructs silico predictions of gene functions using a Multiple Association Network Integration Algorithm [27]. This algorithm is based on linear regression models to calculate functional associations composed of multiple interaction networks and predict gene functions through the constructed network [25,28,29]. The gene functions constructed using this model automatically combine multiple databases that are responsible for creating models that can explain biological connections through genetic interaction networks, shared protein domains, predicted communication, physical interactions, colocalization, and coexpression [25]. The information was consulted on 27 December 2025.

#### 2.9.3. Protein Networking Analysis

From protein–protein interaction networks were constructed using STRING-db [30], the differentially abundant proteins of each group comparison were submitted to Cytoscape (3.10.1) [31] to visualize the protein network plot. The minimum required interaction score was set to medium confidence (0.400), and disconnected nodes were hidden. Network statistics, including the number of nodes, edges, average node degree, clustering coefficient, and PPI enrichment *p*-value, were recorded. MCL (Markov Cluster Algorithm) clustering was applied with an inflation parameter of 1.5 to identify functional modules [32]. Clusters were annotated based on STRING functional enrichment. The information was consulted on 27 December 2025.

#### 2.9.4. Metabolic Networking Analysis

The metabolic pathways were validated by the MetaboAnalyst data module, which encompasses machine learning-based algorithms and robust statistical analyses that enable the generation of high-dimensionality and high-precision data, allowing to explore correlations between metabolic pathways activated in numerous biological conditions, such as osteonecrosis of the jaw [33,34,35]. The information was consulted on 28 December 2025.

#### 2.9.5. Functional Enrichment Analysis

The functional enrichment analysis of proteins commonly identified in the studies included in this review was performed using g:Profiler (https://biit.cs.ut.ee/gprofiler/gost), accessed on 29 December 2025. This functional enrichment platform allows the interaction, mapping, and detection of significantly enriched terms, as well as the analysis of high-throughput genomic data. This analysis platform encompasses data sources from KEGG, WikiPathways, Human Protein Atlas, CORUM, Human Phenotype Ontology, and Gene Ontology. The significance threshold was defined using the Benjamini–Hochberg false discovery rate (FDR) [36], with a *p*-value cutoff of 0.05. The information was consulted on 29 December 2025.

#### 2.9.6. Reactome Analysis

Reactome is an integrative tool that allows users to analyze, visualize, and interpret biomolecular pathways, as well as integrate data from systems biology interpretation, computational modeling, and high-throughput genomic data [37,38]. This platform allows the construction of an interactive panel on hierarchical information in the “Voronoi” style, where the pathways and subpathways related to the process of osteonecrosis of the jaws are displayed with areas proportional to the size of their content and importance in Reactome [39].

### 2.10. Meta-Analysis

Meta-analyses were performed on the R platform (R-4.5, Rstudio extension 2025.05.1+513) using the metafor, dplyr, tibble, stringr, purrr, and readr packages. For each protein, the effect size was the log2 variation between the case group and the control group (log2FC) as a measure of effect size. Proteins with results in at least two studies were included. The logarithmic variation of 2 (log2FC) was calculated. When the standard error was not available, it was assumed that the corresponding two-tailed *p*-values were estimated assuming a standard normal distribution. Only proteins with results in at least two studies were included. These analyses were performed using REML-adjusted random effects models with heterogeneity. They were assessed using the I^2^ statistic and the Q test and analyzed with REML-adjusted random effects models. The results were presented in forest plots [23,40,41,42].

## 3. Results

**Figure 1:**
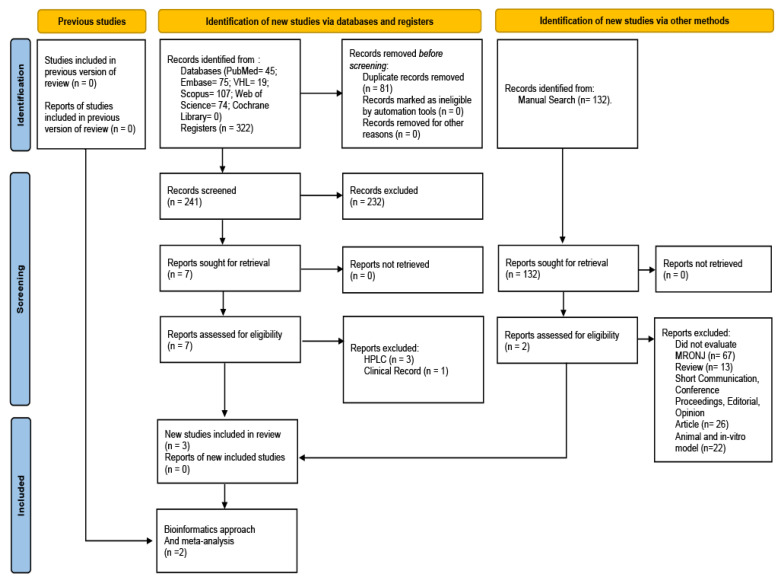
PRISMA flowchart illustrating the study selection and screening process for inclusion in the systematic review and meta-analysis. Studies excluded from the systematic review can be found in Supplementary Material.

Figure 1 represents the synthesis process of the articles identified in the main databases selected in this study. Initially, 322 publications were identified in the PubMed, EMBASE, BVS, Scopus, Web of Science, and Cochrane Library databases. After removing duplicate articles, 241 unique citations were obtained, of which, after applying the eligibility criteria, three publications were obtained that comprised the systematic review and two studies that comprised the meta-analysis. The identification of other methods, such as searching the main journals and gray literature, initially identified 132, of which 67 did not evaluate MRONJ and 22 studies were in vitro. Thus, after additional analysis, the three studies that comprise this systematic review were identified.

Table 1 summarizes the qualitative information extracted from the studies included in this systematic review. It was observed that most of the study designs were case–control studies and that the biological fluid analyzed to verify the proteomic signature was saliva. In addition, the sample size ranged from 10 to 20 individuals per group, aged between the sixth decade of life. Regarding the diagnostic criteria for MRONJ, all studies followed the recommendations of the American Association of Oral and Maxillofacial Surgeons. Regarding the analytical method, high variability was observed among the studies, and the number of proteins identified by these studies ranged from 586 to 1545.

The quality of the data obtained in the studies was assessed using the QUADOMICS tool. All studies present good methodological Quality using the QUADOMICS tool (Supplementary Material).

**Figure 2:**
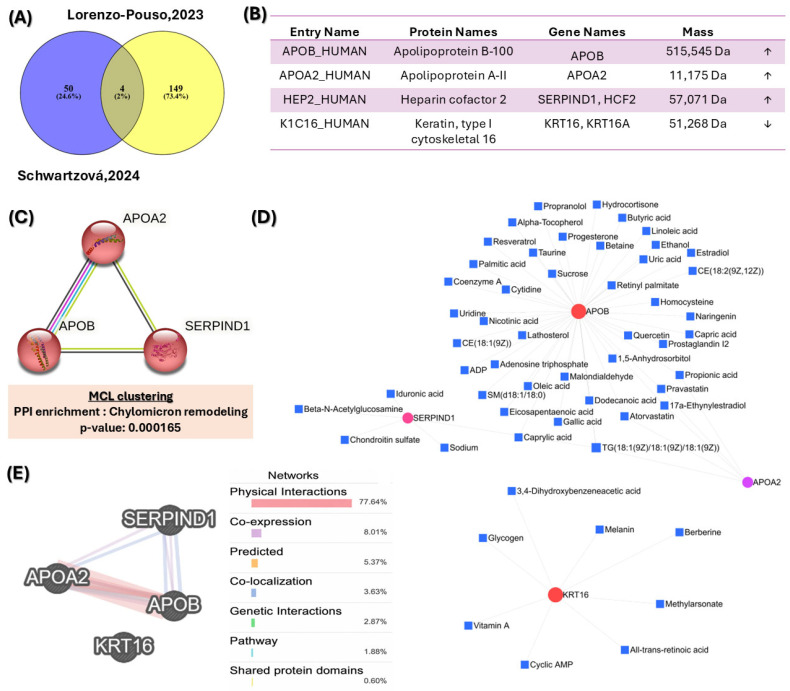
Bioinformatics analyses performed on the proteins identified in the studies included in the systematic review and meta-analysis (**A**) Venn diagram illustrating the distribution and overlap of proteins identified across the included studies [43,44]. (**B**) Lists of information for proteins identified based on UniProt. Upward arrows indicate proteins with increased abundance, whereas downward arrows indicate proteins with decreased abundance in primary proteomic studies. (**C**) Protein–protein interaction network enriched using the bioinformatics approach via STRING. MCL (Markov Cluster Algorithm) clustering was applied with an inflation parameter of 1.5 to identify functional modules. (**D**) Metabolic interaction network using a bioinformatics approach via MetaboAnalyst (Cytoscape program; version 3.10.1). (**E**) Gene-gene interaction network using the bioinformatics approach via GeneMania (Cytoscape program; version 3.10.1).

Figure 2A shows the Venn diagram analysis of differentially abundant proteins in proteomic studies. It can be observed that only 2% (*n* = 4) of the proteins were common to both studies analyzed. Figure 2B represents the information extracted from UniProtKB and the primary studies. It can be observed that the proteins Apolipoprotein B-100 (APOB; 515.54 Da), Apolipoprotein A-II (APOA2; 11.17 Da), and Heparin Cofactor 2 (SERPIND1; 57.07 Da) increased abundance in both studies, while the protein Keratin (KRT16; 51.26 Da) decreased abundance. Figure 2C shows a protein–protein interaction (PPI) network clustered using MCL. The STRING-db network clustering highlighted important functional subgroups, particularly those associated with chylomicron remodeling, with three proteins that were increased abundantly in the studies. Figure 2D characterized the metabolic pathways activated by APOB, APOA2, SERPIND1, and KRT16. We can observe that the APOB protein is responsible for the activation of several metabolic pathways, such as triglycerides, adenosine triphosphate, and progesterone, while the KRT16 protein is responsible for the activation of metabolic pathways such as glycogen, vitamin A, and transretinoic acid. It is observed that the genes encoding the APOA2 and APOB proteins show a higher degree of physical interaction, while the KRT16 protein gene did not connect biologically to the network formed.

**Figure 3:**
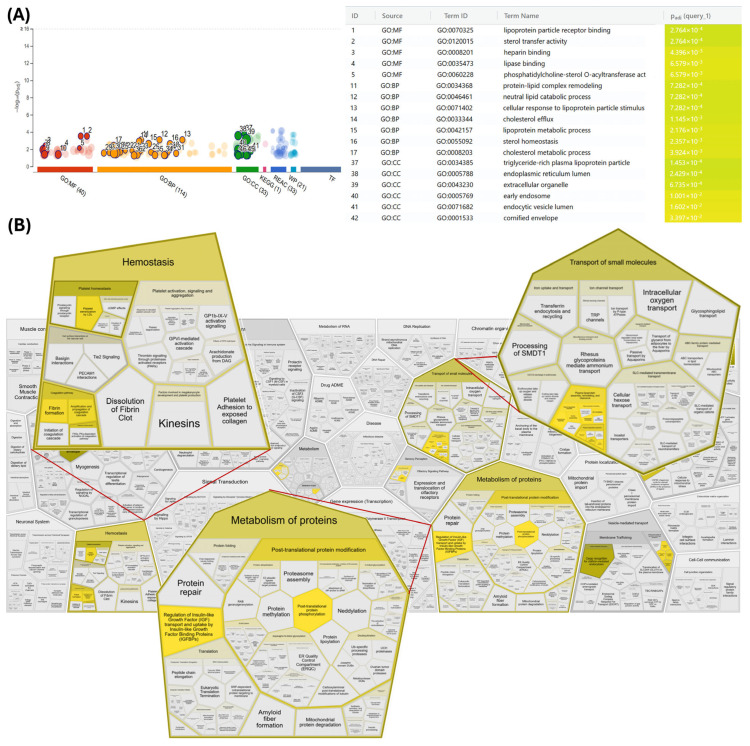
Functional enrichment pathways derived from proteins identified in the primary studies included in the systematic review and meta-analysis. (**A**) Functional enrichment analysis performed using g:Profiler. Significance threshold Benjamini–Hochberg (FDR). *p*-value < 0.05 (Cytoscape program; version 3.10.1). (**B**) Functional enrichment analysis performed using the Reactome database to identify biological pathways associated with the identified molecular targets (Cytoscape program; version 3.10.1).

Figure 3A represents the functional enrichment pathways obtained from proteins commonly identified in the studies and Lorenzo-Pouso et al., 2023 [43] and Schwartzová et al., 2024 [44]. It was observed that the sets of these proteins are responsible for activating the functions GO:MF = “bingin lipoprotein particle receptor (Padj = 3.352 × 10^−4^)” GO:BP = “low-density lipoprotein particle remodeling (Padj = 7.296 × 10^−4^)”, and GO:CC = “triglyceride-rich plasma lipoprotein particle (Padj = 1.454 × 10^−4^)”. Figure 3B represents the pathways enriched by Reactome. It can be observed that this set of proteins plays important roles in homeostasis, small molecule transport, and protein metabolism.

**Figure 4:**
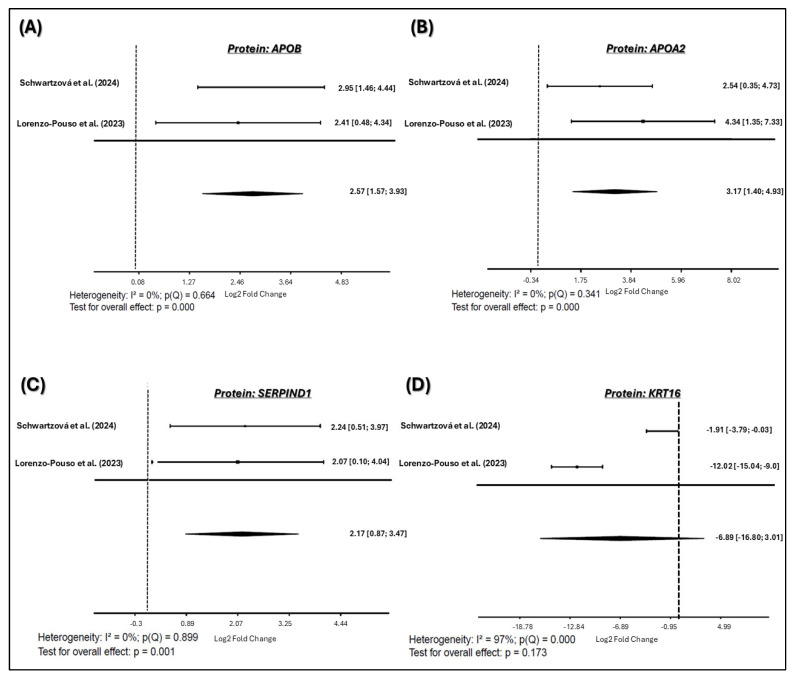
Forest plot of the meta-analysis evaluating proteins with differential abundance in the saliva of patients with MRONJ compared with healthy individuals [43,44]. (**A**) Meta-analysis of the APOB protein; (**B**) Meta-analysis of the APOA2 protein; (**C**) Meta-analysis of the SERPIND1 protein; (**D**) Meta-analysis of the KRT16 protein. Squares represents the effect estimates of individual studies, with horizontal lines indicating the corresponding 95% confidence intervals. Diamonds represent the pooled overall effect size for each protein, with numerical values expressed as log_2_ fold change and 95% confidence intervals. Heterogeneity statistics, including I^2^ and *p*(Q), and the test for overall effect are shown in each panel. Statistical significance was set at *p* < 0.05.

The results obtained in our meta-analysis can be seen in Figure 4. We can see that the proteins APOB [2.75-95% CI 1.57 to 3.93; *p* < 0.0001), APOA2 [3.17-95% CI 1.40 to 4.93; *p* < 0.0001), and SERPIND1 [2.17-95% CI 0.87 to 3.47; *p* < 0.001] were significantly increased in the saliva of patients with MRONJ. In contrast, the protein KRT16 [−6.89-95% CI −16.80 to 3.01; *p* = 0.173] did not show a significant association, although it showed decreased abundance in the saliva of patients with MRONJ.

## 4. Discussion

To the best of our knowledge, this is the first study to perform a comprehensive proteomic meta-analysis supported by robust bioinformatic approaches in individuals affected by MRONJ. This systematic review highlights the potential role of novel proteomic biomarkers in the clinical monitoring of osteonecrosis of the jaw. Although extensively investigated, the pathophysiology and molecular mechanisms underlying this condition remain unclear [46]. The narrative results (Table 1) showed that saliva was the most frequently investigated biological fluid for identifying proteomic signature profiles; in fact, it was the exclusive fluid analyzed in the eligible studies. Therefore, it should be explicitly noted that no studies utilizing serum or tissue samples were included in our review, nor did the evaluated literature explore protein isoforms (proteoforms) in these tissues. The analysis of salivary proteins as a non-invasive method offers a low-risk and cost-effective strategy for monitoring various diseases [27], including MRONJ. The results of our meta-analysis (Figure 2B) revealed significantly elevated levels of APOA2, APOB, and SERPIND1 proteins in the saliva of patients with MRONJ. In contrast, KRT16 levels decreased, although this did not reach statistical significance.

Regarding the salivary proteomic profile of individuals affected by MRONJ, we identified a reduced abundance of KRT16. To our knowledge, based on a systematic and comprehensive review of the literature, this is the first study to highlight reduced protein signatures in the saliva of individuals with MRONJ. KRT16 is an important type I cytoskeletal protein that is integrated into a genetic regulatory network involved in danger signaling [47,48], activation of the innate immune system, modulation of epidermal barrier function, and regulation of damage-associated molecular patterns (DAMPs) by keratinocytes [49,50,51]. Current evidence indicates that keratinocytes exposed to stress conditions such as injury, inflammation, or intense environmental stress may upregulate the transcription of keratin-associated factors such as KRT6, KRT16, and KRT17 in the oral mucosa [52]. Changes in the expression pattern of these transcriptional factors generally occur at the expense of physiological keratin differentiation (KRT1/KRT10), temporarily delaying terminal differentiation to promote cell migration and support local tissue repair processes [53].

Several studies suggest that antiresorptive agents and monoclonal antibodies do not exert direct effects on intact connective tissue [54,55,56]. However, in the presence of tissue injury or inflammation, osteoclast activity is suppressed, delaying bone healing [9], which may result in persistent inflammation or infection [57], soft tissue toxicity [58], inhibition of angiogenesis [8], and immune system dysfunction [45,59]. Thus, inflammation and infection associated with osteonecrosis may disrupt the production of physiological markers, such as KRT16. Patients with MRONJ exhibit chronic, dysregulated inflammation associated with impaired cellular repair mechanisms, which may impair epithelial adaptive capacity, leading to decreased abundance of protective proteins, including KRT16. This protein plays a critical role in maintaining cellular resilience and mechanical integrity, enabling keratinocytes to withstand physical stress during the wound healing process [45,46,53,60].

Other proteins that exhibited statistically significant alterations in our meta-analysis included APOA2 and APOB. Human APOA2 is a glycosylated protein synthesized predominantly in the liver and, to a lesser extent, in the intestine [61]. As the second most abundant apolipoprotein in high-density lipoprotein particles, it plays an important role in the dynamic regulation of lipid metabolism, neutrophil cytokine production, and inflammatory stress responses [62,63]. Its lipid-binding properties are related to amphipathic helical regions, structural features typical of apolipoproteins involved in lipoprotein remodeling [64,65]. Previous studies by Lorenzo-Pouso et al., 2023 [43] and Schwartzová et al., 2024 [44] reported significantly higher differential abundance of APOA2 in individuals with MRONJ, compared with clinically healthy individuals [46,64]. The overproduction of lipoproteins and fibrinogen in MRONJ may be associated with altered fibrinolytic activity and coagulation disorders (thrombophilia), potentially increasing the risk of local ischemia and, consequently, jawbone necrosis [66]. Notably, bioinformatic analyses conducted by our research group revealed enriched pathways associated with lipid metabolism, including direct metabolic associations with triglyceride levels and APOA (Figure 3B), as well as MCL clustering PPI enrichment related to chylomicron remodeling (Figure 2C). Significant functional enrichment was also identified for GO:0070325-Lipoprotein particle receptor binding and GO:0042157-Lipoprotein metabolic process (Figure 3A). Additionally, enrichment was observed in pathways related to hemostasis, including platelet sensitization by LDL, formation of the fibrin clot (clotting cascade), and the intrinsic pathway of fibrin clot formation (Figure 3B).

APOA2 has also been reported to exert pro-inflammatory effects by enhancing monocyte responses to lipopolysaccharide (LPS) [67]. Its functions involve modulation of LPS responses through direct binding to LPS or by altering the responsiveness of LPS-sensitive cells [68]. Therefore, alterations in apolipoprotein abundance, particularly APOA2, may contribute to the amplification and persistence of inflammatory responses in chronic bacterial infection settings such as MRONJ [45,46,60].

Conversely, APOB demonstrated an inverse correlation with bone mineral density (BMD), indicating that individuals with elevated levels of this protein may be at increased risk of osteoporosis or osteopenia [69,70,71]. APOB is an important structural apolipoprotein of chylomicrons that acts as a recognition signal for LDL receptor-mediated internalization. From a structure-function perspective, APOB contains amphipathic regions of α-helices and β-sheets associated with lipids, which support its role in lipoprotein assembly and lipid transport [72,73]. Furthermore, recent epidemiological and clinical studies have reported a strong association between lipid biomarkers and BMD [71,72,73,74,75,76,77,78]. Although the mechanism underlying this association remains uncertain, hypotheses suggest that oxidized lipids and apolipoproteins may directly impair bone cells by inhibiting osteoblast-mediated bone matrix deposition and promoting osteoclastic activity, resulting in reduced BMD [79,80]. These findings suggest that APOB may have potential as a biomarker for individuals with low BMD and MRONJ. However, the biological role of this protein remains incompletely understood, and further population-based studies are needed.

One of the proteins found to be increased in abundance in our analyses was SERPIND1, also known as heparin cofactor II, a serine protease that acts as a potent and selective inhibitor of thrombin [81]. From a structure-function perspective, SERPIND1 belongs to the serpin family and contains conserved structural features that support its role in protease inhibition [82,83]. Under physiological conditions, this protein plays a critical role in modulating the coagulation cascade by limiting excessive fibrin formation following vascular injury, and promoting tissue repair [46,81,82,83]. French et al., 2008 [84] reported a 2.79-fold increased risk of osteonecrosis in individuals carrying the PAI-1 gene polymorphism (SERPIND1, rs6092) characterized by a 4G/5G insertion/deletion repeat in the promoter region [85]. Additionally, the presence of the 4G allele has been associated with several diseases, including acute myocardial infarction, atherosclerosis, and metabolic syndrome. Notably, our bioinformatic analyses revealed that APOA2, APOB, and SERPIND1 exhibit strong physical interaction (77.6%) and co-expression (8%) (Figure 2E) and are concurrently involved in key metabolic pathways associated with triglyceride metabolism (Figure 2D). As described by Schwartzová et al., 2024 [44], the central role of SERPIND1 in MRONJ pathogenesis may involve the facilitation of wound healing processes and modulation of thrombin-mediated chemotactic and mitogenic activities [86]. In this context, SERPIND1 may serve as a potential biomarker reflecting healing responses in medication-related necrotic alterations in MRONJ patients.

Elevated levels of this protein have also been reported in skin healing contexts associated with radiotherapy, suggesting a role in vascular injury responses [84]. Moreover, partial degradation by inflammatory proteases, SERPIND1, may generate fragments with chemotactic activity toward neutrophils and monocytes, thereby contributing to the modulation of local inflammatory responses [81,83,86,87,88,89,90,91]. Therefore, in pathological contexts characterized by persistent lesions, chronic inflammation, and impaired healing, such as MRONJ, increased SERPIND1 abundance may be interpreted not as a primary etiological factor, but rather as a component of adaptive responses to tissue damage [45,46,60].

Finally, this study presents several limitations. A major constraint is the limited availability of literature investigating proteomic approaches for biomarker identification in MRONJ. Although three studies met the eligibility criteria for systematic review, only two [46,64] were suitable for inclusion in bioinformatic and quantitative (meta-analytic) analyses. Furthermore, methodological heterogeneity was observed regarding participant populations (different countries), sex, age, and proteomic platforms. The interpretation of the meta-analysis results must also take statistical heterogeneity into account. Although low I^2^ values were observed for some proteins, the data indicate substantial heterogeneity for KRT16, reflecting the variability among the studies included. Furthermore, the handling of missing values represents a significant methodological limitation, as strategies for dealing with missing data and normalization procedures were not consistently reported in the primary studies. Since this review is based on published data, no additional imputation was performed. The potential impact of risk of bias must also be considered, as the small number of eligible studies precludes formal subgroup analyses based on methodological quality. The study by Thumbigere-Math et al., 2015 [13] was excluded from these analyses due to the use of bisphosphonate-treated, disease-free patients as controls instead of clinically healthy individuals, potentially introducing confounding factors. This methodological limitation prevented the inclusion of this study in our meta-analysis and bioinformatic analyses, which might otherwise have increased the robustness of identifying MRONJ proteomic signatures. Another important partial limitation is that our study did not explore proteoforms, protein species, or broader proteome complexity using dedicated proteomic databases or proteoform-centered analytical strategies. Instead, the main objective was to identify eligible proteomic studies and, based on the abundance data of each protein reported in each individual study, perform integrated bioinformatic combinations and meta-analyses. In this respect, our methodological pathway followed the strategy proposed by Ahmad et al., 2025 [23], prioritizing the integration of published proteomic findings rather than direct reanalysis of raw proteomic datasets or characterization of proteoform diversity. The use of data available in omics databases also presented challenges, as protein datasets and included studies were relatively small and derived from previously published datasets from the general medical literature. Also, saliva was the exclusive fluid analyzed in the eligible studies. Therefore, it should be explicitly noted that no studies utilizing serum or tissue samples were included in our review, nor did the evaluated literature explore protein isoforms (proteoforms). Given these constraints, our findings should be interpreted with caution and considered preliminary and require further validation in larger, independent cohorts. Despite these limitations, this study provides a clear and objective contribution to the exploratory identification of uncommon proteins that may serve as therapeutic targets and diagnostic biomarkers in MRONJ.

## 5. Conclusions

This systematic review and meta-analysis, supported by bioinformatics approaches, identified a distinct salivary proteomic profile associated with medication-related osteonecrosis of the jaw (MRONJ). The increased levels of APOB, APOA2, and SERPIND1 in affected patients, together with the trend toward reduced KRT16 abundance, suggest that disruptions in lipid metabolism, tissue repair, and immune-inflammatory pathways play a role in MRONJ pathophysiology. Despite the limited number of included studies and the inherent heterogeneity, these findings provide exploratory evidence of potential salivary biomarkers and contribute to a better understanding of the molecular mechanisms underlying the disease. Specifically, the identification of these non-invasive salivary biomarkers provides a promising framework for the screening of high-risk populations, such as patients undergoing long-term antiresorptive therapy. Furthermore, they could serve as practical tools for clinical and treatment monitoring, allowing for the early detection of disease onset and the evaluation of therapeutic efficacy over time.

Further well-designed, large-scale studies are warranted to validate these biomarkers and to elucidate their clinical applicability in early diagnosis, risk assessment, and therapeutic targeting of MRONJ.

## Figures and Tables

**Table 1:** Summary of information obtained for systematic review.

Author/Year	Study Design/Sample Medium/ Sample Size	Demographic Profile Case/Control	Diagnostic Criteria: Case	Diagnostic Criteria: Control	Stage	Type of Medication	Analysis Method (Model)	Number of Proteins Identified/ Bioinformatics Approach
Lorenzo-Pouso, 2023 [43]	Case–control; Saliva; 18 cases/ 20 controls	Case: Gender: 14 women; 4 men Age: 69.8 ± 9.5 years Control: Gender: None Age: 71.8 years	All patients had been diagnosed with MRONJ: American Association of Oral and Maxillofacial Surgeons [2]	Healthy individuals without systemic or other diseases	Stage 1: 15 cases (83.3%); stage 2: 2 cases (11.1%); and stage 3: 1 case (6.6%)	Intravenous zoledronate (*n* = 12 patients); oral pamidronate (*n* = 2 patients), and subcutaneous denosumab (*n* = 4 patients)	Quadrupole-TOF (Q-TOF)—TripleTOF 6600 (SCIEX, Marlborough, MA, USA)	Identification of 586 proteins/Yes
Schwartzová, 2024 [44]	Case–control; Saliva; 10 cases/ 10 controls	Case: Gender: 8 women; 2 men Age: 67.6 ± 7.2 years Control: Gender: 6 women; 4 men Age: 29.0 ± 9.8 years	All patients had been diagnosed with MRONJ: American Association of Oral and Maxillofacial Surgeons [2]	Healthy individuals without systemic or other diseases	The study was limited to participants with necrotic bone exposed to the oral cavity, corresponding to stages 1 through 3, as defined by the classification system.	Intravenous zoledronic acid (*n* = 6 patients); Intravenous pamidronic acid (*n* = 2 patients), and intravenous ibandronic acid (*n* = 2 patients)	Orbitrap Eclipse Tribrid + nanoLC Ultimate 3000 (Thermo Fisher Scientific, Waltham, MA, USA)	Identification of 1545 proteins/Yes
Thumbigere-Math, 2015 [13]	Case–control; Saliva; 20 cases/ 20 controls	Case: Gender: 13 women; 7 men Age: 64.3 ± 10.1 years Control: Gender: 17 women; 3 men Age: 62.4 ± 11.3 years	All patients had been diagnosed with MRONJ: American Association of Oral and Maxillofacial Surgeons [45]	Control patients who did not develop MRONJ following intravenous bisphosphonate therapy	Control: stage 0; Case: not informed	Control: Intravenous zoledronate (*n* = 18 patients); Intravenous Pamidronate + Zoledronate (*n* = 2 patients) Case: Intravenous zoledronate (*n* = 14 patients); Intravenous pamidronate (*n* = 2 patients) Intravenous pamidronate + zoledronate (*n* = 4 patients)	LTQ-Orbitrap Velos (Thermo Fisher Scientific, Waltham, MA, USA)	Identification of 1340 proteins/Yes

## Data Availability

The data that support the findings of this study are available from the corresponding author upon reasonable request.

## Supplementary Materials

The following supporting information can be downloaded at https://www.mdpi.com/article/10.3390/proteomes14020029/s1, Table S1: Strategic search in selected databases; Table S2: List of studies excluded from the systematic review; Table S3: Quality analysis of the studies included in the systematic review using the QUADOM-ICS tool.
