# Supplementary material for: Systematic Review of Protein Signatures for Clinical Monitoring of Osteonecrosis of the Jaw: Meta-Analysis and Insights from Bioinformatics-Driven Proteomics
Source: Proteomes. 2026 Jun 10;14(2):29. doi: 10.3390/proteomes14020029 (PMC13306536; doi:10.3390/proteomes14020029)
Supplement: Supplementary file 1 [file proteomes-14-00029-s001.zip › proteomes-4241612-supplementary.pdf]

proteomes

MDPI

Supplementary Material

Table S1. Strategic search in selected databases.

**PubMed: #1 AND #2 = 45 Articles [All Fields]**
#1= ((((((((((((((((((((("bisphosphonate associated osteonecrosis of the jaw") OR ("bisphosphonate related osteonecrosis of the jaw")) OR ("bisphosphonate associated")) OR ("bisphosphonate-associated osteonecrosis of the jaw")) OR ("bisphosphonate related osteonecrosis of the jaw")) OR ("bisphosphonate osteonecrosis")) OR ("bisphosphonate induced osteonecrosis of the jaw")) OR ("BRONJ")) OR ("BONJ")) OR ("MRONJ")) OR ("ARONJ")) OR ("DRONJ")) OR ("avascular necrosis of the jaw")) OR ("jaw necrosis")) OR ("mandibular osteonecrosis")) OR ("maxillary osteonecrosis")) OR ("zoledronic acid osteonecrosis")) OR ("alendronate osteonecrosis")) OR ("pamidronate osteonecrosis")) OR ("risedronate osteonecrosis")) OR ("ibandronate osteonecrosis")) OR ("clodronate osteonecrosis")<br>#2= (((((((((((((((((("MS") OR ("MS/MS")) OR ("MS-MS")) OR ("LC/MS")) OR ("LC-MS/MS")) OR ("MALDI-TOF")) OR ("MALDI-TOF-MS")) OR ("SELDI-TOF")) OR ("SELDI-TOF-MS")) OR ("MALDI")) OR ("SELDI")) OR ("mass spectrometry")) OR ("liquid chromatography")) OR ("matrix-assisted laser desorption/ionization")) OR ("surface-enhanced laser desorption/ionization")) OR ("time-of-flight")) OR ("proteomics")) OR ("proteomic")) OR ("proteomic analysis")
**EMBASE: #1 AND #2 = 75 Articles [All Fields]**
#1= ('bisphosphonate-associated osteonecrosis of the jaw' OR 'bisphosphonate-associated osteonecrosis of the jaw' OR 'bisphosphonate-related osteonecrosis of the jaw' OR 'bisphosphonate induced osteonecrosis of the jaw' OR 'bisphosphonate osteonecrosis' OR 'bisphosphonate associated' OR 'BRONJ' OR 'BONJ' OR 'MRONJ' OR 'ARONJ' OR 'DRONJ' OR 'avascular necrosis of the jaw' OR 'jaw necrosis' OR 'mandibular osteonecrosis' OR 'maxillary osteonecrosis' OR 'zoledronic acid osteonecrosis' OR 'alendronate osteonecrosis' OR 'pamidronate osteonecrosis' OR 'risedronate osteonecrosis' OR 'ibandronate osteonecrosis' OR 'clodronate osteonecrosis')<br>#2= ('MS' OR 'MS/MS' OR 'MS-MS' OR 'LC/MS' OR 'LC-MS/MS' OR 'MALDI-TOF' OR 'MALDI-TOF-MS' OR 'SELDI-TOF' OR 'SELDI-TOF-MS' OR 'MALDI' OR 'SELDI' OR 'mass spectrometry' OR 'liquid chromatography' OR 'matrix-assisted laser desorption/ionization' OR 'surface-enhanced laser desorption/ionization' OR 'time-of-flight' OR 'proteomics' OR 'proteomic' OR 'proteomic analysis')
**Scopus: 107 Articles [All Fields]**
("MS" OR "MS/MS" OR "MS-MS" OR "LC/MS" OR "LC-MS/MS" OR "MALDI-TOF" OR "MALDI-TOF-MS" OR "SELDI-TOF" OR "SELDI-TOF-MS" OR "MALDI" OR "SELDI" OR "mass spectrometry" OR "liquid chromatography" OR "matrix-assisted laser desorption/ionization" OR "surface-enhanced laser desorption/ionization" OR "time-of-flight" OR "proteomics" OR "proteomic" OR "proteomic analysis") AND ('bisphosphonate-associated osteonecrosis of the jaw' OR 'bisphosphonate-related osteonecrosis of the jaw' OR 'bisphosphonate induced osteonecrosis of the jaw' OR 'bisphosphonate osteonecrosis' OR 'bisphosphonate associated' OR 'BRONJ' OR 'BONJ' OR 'MRONJ' OR 'ARONJ' OR 'DRONJ' OR 'avascular necrosis

 https://doi.org/10.3390/proteomes14020029

of the jaw' OR 'jaw necrosis' OR 'mandibular osteonecrosis' OR 'maxillary osteonecrosis' OR 'zoledronic acid osteonecrosis' OR 'alendronate osteonecrosis' OR 'pamidronate osteonecrosis' OR 'risedronate osteonecrosis' OR 'ibandronate osteonecrosis' OR 'clodronate osteonecrosis')
**Cochrane Library: 0 Articles**
Title/Abstract/keyword: ("bisphosphonate-associated osteonecrosis of the jaw" OR "bisphosphonate-related osteonecrosis of the jaw" OR "bisphosphonate induced osteonecrosis of the jaw" OR "bisphosphonate osteonecrosis" OR "bisphosphonate associated" OR "BRONJ" OR "BONJ" OR "MRONJ" OR "ARONJ" OR "DRONJ" OR "avascular necrosis of the jaw" OR "jaw necrosis" OR "mandibular osteonecrosis" OR "maxillary osteonecrosis" OR "zoledronic acid osteonecrosis" OR "alendronate osteonecrosis" OR "pamidronate osteonecrosis" OR "risedronate osteonecrosis" OR "ibandronate osteonecrosis" OR "clodronate osteonecrosis")<br>Title/Abstract/keyword:("MS" OR "MS/MS" OR "MS-MS" OR "LC/MS" OR "LC-MS/MS" OR "MALDI-TOF" OR "MALDI-TOF-MS" OR "SELDI-TOF" OR "SELDI-TOF-MS" OR "MALDI" OR "SELDI" OR "mass spectrometry" OR "liquid chromatography" OR "matrix-assisted laser desorption/ionization" OR "surface-enhanced laser desorption/ionization" OR "time-of-flight" OR "proteomics" OR "proteomic" OR "proteomic analysis")
**VHL= 19 Articles**
("bisphosphonate-associated osteonecrosis of the jaw" OR "bisphosphonate-related osteonecrosis of the jaw" OR "bisphosphonate induced osteonecrosis of the jaw" OR "bisphosphonate osteonecrosis" OR "bisphosphonate associated" OR "BRONJ" OR "BONJ" OR "MRONJ" OR "ARONJ" OR "DRONJ" OR "avascular necrosis of the jaw" OR "jaw necrosis" OR "mandibular osteonecrosis" OR "maxillary osteonecrosis" OR "zoledronic acid osteonecrosis" OR "alendronate osteonecrosis" OR "pamidronate osteonecrosis" OR "risedronate osteonecrosis" OR "ibandronate osteonecrosis" OR "clodronate osteonecrosis") AND ("MS" OR "MS/MS" OR "MS-MS" OR "LC/MS" OR "LC-MS/MS" OR "MALDI-TOF" OR "MALDI-TOF-MS" OR "SELDI-TOF" OR "SELDI-TOF-MS" OR "MALDI" OR "SELDI" OR "mass spectrometry" OR "liquid chromatography" OR "matrix-assisted laser desorption/ionization" OR "surface-enhanced laser desorption/ionization" OR "time-of-flight" OR "proteomics" OR "proteomic" OR "proteomic analysis")
**WEB OF SCIENCE: 74 Articles**
("bisphosphonate-associated osteonecrosis of the jaw" OR "bisphosphonate-related osteonecrosis of the jaw" OR "bisphosphonate induced osteonecrosis of the jaw" OR "bisphosphonate osteonecrosis" OR "bisphosphonate associated" OR "BRONJ" OR "bone" OR "MRONJ" OR "arons" OR "drone" OR "avascular necrosis of the jaw" OR "jaw necrosis" OR "mandibular osteonecrosis" OR "maxillary osteonecrosis" OR "zoledronic acid osteonecrosis" OR "alendronate osteonecrosis" OR "pamidronate osteonecrosis" OR "risedronate osteonecrosis" OR "ibandronate osteonecrosis" OR "clodronate osteonecrosis") (All Fields) and ("MS" OR "MS/MS" OR "MS-MS" OR "LC/MS" OR "LC-MS/MS" OR "MALDI-TOF" OR "MALDI-TOF-MS" OR "SELDI-TOF" OR "SELDI-TOF-MS" OR "MALDI" OR "SELDI" OR "mass spectrometry" OR "liquid chromatography" OR "matrix-assisted laser desorption/ionization" OR "surface-enhanced laser desorption/ionization" OR "time-of-flight" OR "proteomics" OR "proteomic" OR "proteomic analysis") (All Fields)

https://doi.org/10.3390/xxxxx

Table S2. List of studies excluded from the systematic review.

	Name/year	Title of Study	Reason
		**Databases**	
	Kim et al. (2017)	Wound healing protein profiles in the postoperative exudate of bisphosphonate-related osteonecrosis of mandible	HPLC analysis
2	Kim et al. (2018)	Immunoprecipitation high performance liquid chromatographic analysis of healing process in chronic suppurative osteomyelitis of the jaw	HPLC analysis
3	Kolokythas et al. (2015)	Salivary biomarkers associated with bone deterioration in patients with medication-related osteonecrosis of the jaws	HPLC analysis
4		Bone Healing After Dental Extraction in Postmenopausal Osteoporotic Women Treated With Alendronate Per os (70 mg Weekly)	Clinical Record
		**Manual Search**	
5	Dou et al. (2018)	iTRAQ-Based Proteomic Analysis Exploring the Influence of Hypoxia on the Proteome of Dental Pulp Stem Cells under 3D Culture	In vitro
6	Spreafico et al. (2006)	A proteomic study on human osteoblastic cells proliferation and differentiation	In vitro
7	Bivi et al. (2011)	Shotgun proteomics analysis reveals new unsuspected molecular effectors of nitrogen-containing bisphosphonates in osteocytes	In vitro
8	Nakai et al. (2024)	Use of data-independent acquisition mass spectrometry to identify an objective serum indicator of the need for osteoporotic therapeutic intervention	Does not address MRONJ
9	Hoover et al. (2020)	Proteomic characterization of a trauma-based rat model of heterotopic ossification identifies interactive signaling networks as potential therapeutic targets	Animal model
10	Csősz et al. (2017)	Quantitative body fluid proteomics in medicine — A focus on minimal invasiveness	Review
11	López-Cortés et al. (2021)	Blood-based protein biomarkers in bladder urothelial tumors	Review
12	Chen et al. (2020)	Quantitative proteomics and reverse engineer analysis identified plasma exosome derived protein markers related to osteoporosis.	Does not address MRONJ
13	Bivi et al. (2009)	Transcriptome and Proteome Analysis of Osteocytes Treated with Nitrogen-Containing Bisphosphonates	In vitro

https://doi.org/10.3390/xxxxx

14	Ihling et al. (2015)	Acidosis-Induced Changes in Proteome Patterns of the Prostate Cancer-Derived Tumor Cell Line AT-1	In vitro
15	Gibson et al. (2006)	Proteomic Analysis of Recurrent Joint Inflammation in Juvenile Idiopathic Arthritis	Does not address MRONJ
16	Zhu et al. (2015)	A Comprehensive Proteomics Analysis Reveals a Secretory Path- and Status-Dependent Signature of Exosomes Released from Tumor-Associated Macrophages	In vitro
17	Dumont et al. (2012)	Differential proteomic analysis of a human breast tumor and its matched bone metastasis identifies cell membrane and extracellular proteins associated with bone metastasis	Does not address MRONJ
18	Kessler et al. (2020)	Enrichment of Collagen Fragments Using Dimeric Collagen Hybridizing Peptide for Urinary Collagenomics<br>Using an In-Sample Addition of Medronic Acid for the Analysis of Purine- and Pyrimidine-Related	Does not address MRONJ
19	Lin et al. (2023)	Using an In-Sample Addition of Medronic Acid for the Analysis of Purine- and Pyrimidine-Related Derivatives and Its Application in the Study of Lung Adenocarcinoma A549 Cell Lines by LC–MS/MS	Does not address MRONJ
20	De Lima-Souza et al. (2022)	Risks And Benefits Of Bisphosphonates: Are Patients Well Informed?	Does not address MRONJ
21	Costa et al. (2022)	Global Proteomic Profiling Reveals Metabolic Reprogramming In Ameloblastomas	Does not address MRONJ
22	Martelli et al. (2022)	Analysis Of Oral Medicine And Oral Pathology Teaching In Higher Education In Brazil	Does not address MRONJ
23	Balbinot, Loureiro et al. (2022)	Immunoexpression Of Stem Cell Markers Sox-2, Nanog, And Oct4 In Ameloblastoma	Does not address MRONJ
24	Balbinot, Da Silva, et al. (2022)	Odontogenic Glandular Cyst: A Collaborative Investigation Of 22 Cases And Proteins Related To Invasiveness	Does not address MRONJ
		**Grey Literature (Google Scholar)**	
25	Bivi et al. (2009)	Transcriptome and proteome analysis of osteocytes treated with nitrogen-containing bisphosphonates	In vitro

26	Bivi et al. (2011)	Shotgun proteomics analysis reveals new unsuspected molecular effectors of nitrogen-containing bisphosphonates in osteocytes	In vitro
27	J. Kim et al. (2021)	Alendronate-induced perturbation of the bone proteome and microenvironmental pathophysiology	In vitro
28	Romanello et al. (2014)	Osteoblastic cell secretome: a novel role for progranulin during risedronate treatment	In vitro
29	Ilyas et al. (2014)	The effect of alendronate on proteome of hepatocellular carcinoma cell lines	In vitro
30	Cinier et al. (2009)	Bisphosphonate adaptors for specific protein binding on zirconium phosphonate-based microarrays	In vitro
31	Kaps et al. (2022)	pH-degradable, bisphosphonate-loaded nanogels attenuate liver fibrosis by repolarization of M2-type macrophages	In vitro
32	Romanello et al., 2006	Bisphosphonates activate nucleotide receptors signaling and induce the expression of Hsp90 in osteoblast-like cell lines	In vitro
33	Plotkin et al. (2005)	Bisphosphonates and estrogens inhibit osteocyte apoptosis via distinct molecular mechanisms downstream of extracellular signal-regulated kinase activation	In vitro
34	Reszka et al., (1999)	Bisphosphonates Act Directly on the Osteoclast to Induce Caspase Cleavage of Mst1 Kinase during Apoptosis: A LINK BETWEEN INHIBITION OF THE MEVALONATE PATHWAY AND REGULATION OF AN APOPTOSIS-PROMOTING KINASE	In vitro
35	Yan et al. (2025)	Using network pharmacology and molecular docking technology, proteomics and experiments were used to verify the effect of Yigu decoction (YGD) on the expression of key genes in osteoporotic mice	Animal model
36	Alaiya et al. (2014)	Proteomic analysis of soft tissue tumor implants treated with a novel polybisphosphonate	Animal model
37	Zhao et al. (2007)	Effects of hindlimb unloading and bisphosphonates on the serum proteome of rats	Animal model
38	Su et al. (2024)	Protective mechanism of alendronate granule in a rat osteoporosis model based on TMT proteomic analysis	Animal model

39	Hoover et al. (2020)	Proteomic characterization of a trauma-based rat model of heterotopic ossification identifies interactive signaling networks as potential therapeutic targets	Animal model
40	Funayama et al. (2023)	Impact of beta-tricalcium phosphate on preventing tooth extraction-triggered bisphosphonate-related osteonecrosis of the jaw in rats	Animal model
41	Yang et al. (2022)	TMT-based proteomics analysis to screen potential biomarkers of Achyranthis Bidentatae Radix for osteoporosis in rats	Animal model
42	Ali et al. (2015)	A highly sensitive prenylation assay reveals in vivo effects of bisphosphonate drug on the Rab prenylome of macrophages outside the skeleton	Animal model
43	Z. Yan et al., 2025	Bisphosphonate-mineralized nano-IFNγ suppresses residual tumor growth caused by incomplete radiofrequency ablation through metabolically remodeling tumor-associated macrophages	Animal model
44	Nielson et al. (2017)	Identification of hip BMD loss and fracture risk markers through population-based serum proteomics	Does not address MRONJ
45	Kawada et al. (2025)	Serum proteomic profiles of patients with chronic recurrent multifocal osteomyelitis	Does not address MRONJ
46	Chen et al. (2020)	Quantitative proteomics and reverse engineer analysis identified plasma exosome derived protein markers related to osteoporosis.	Does not address MRONJ
47	Dai et al. (2025)	Integrating thermal proteome profiling and virtual screening to reveal the mechanism of Bletilla striata against osteoclast-driven osteoporosis	Does not address MRONJ
48	Chaput et al. (2012)	A proteomic study of protein variation between osteopenic and age-matched control bone tissue	Does not address MRONJ
49	Dowling et al. (2014)	Identification of proteins found to be significantly altered when comparing the serum proteome from Multiple Myeloma patients with varying degrees of bone disease	Does not address MRONJ
50	Wang et al. (2024)	Proteomic insights into osteoporosis: unraveling diagnostic markers of and therapeutic targets for the metabolic bone disease	Does not address MRONJ
51	Onono et al. (2009)	A Novel Proteomic Approach to Define Leukemia Cell Resistance to Farnesyltransferase Inhibitors.	Does not address MRONJ

https://doi.org/10.3390/xxxxx

52	Hasebe et al. (2025)	Leveraging Proteomics and Proteogenomics for Understanding Osteoporosis and Other Musculoskeletal Diseases	Does not address MRONJ
53	Kuo et al. (2015)	Complement component C3: Serologic signature for osteogenesis imperfecta. Analysis of a comparative proteomic study	Does not address MRONJ
54	Fellows et al. (2016)	Applying proteomics to study crosstalk at the cartilage-subchondral bone interface in osteoarthritis: current status and future directions	Does not address MRONJ
55	Wood et al. (2022)	Identification of new therapeutic targets of bone cancers by proteomic strategies	Does not address MRONJ
56	Jahnke et al. (2015)	A general strategy for targeting drugs to bone	Does not address MRONJ
57	Conti et al. (2011)	Identification of potential biomarkers for giant cell tumor of bone using comparative proteomics analysis	Does not address MRONJ
58	Sun et al. (2018)	Genomic atlas of the human plasma proteome	Does not address MRONJ
59	Csosz et al. (2017)	Proteomics investigation of OSCC-specific salivary biomarkers in a Hungarian population highlights the importance of identification of population-tailored biomarkers	Does not address MRONJ
60	Iglesias-Gato et al. (2018)	The proteome of prostate cancer bone metastasis reveals heterogeneity with prognostic implications	Does not address MRONJ
61	Ilyas et al. (2015)	Effects of 5′-azacytidine and alendronate on a hepatocellular carcinoma cell line: a proteomics perspective	Does not address MRONJ
62	Hawash et al. (2023)	Proteomic analysis of chronic wound exudate reveals spatially localized predictive and diagnostic biomarkers of healing outcome	Does not address MRONJ
63	Ho et al. (2020)	Proteomics-inspired precision medicine for treating and understanding multiple myeloma	Does not address MRONJ
64	Sanz-Rodríguez et al. (2007)	Bisphosphonates as inhibitors of Trypanosoma cruzi hexokinase: kinetic and metabolic studies	Does not address MRONJ
65	Ho et al. (2023)	An atlas of the bone marrow bone proteome in patients with dysproteinemias	Does not address MRONJ

https://doi.org/10.3390/xxxxx

66	Qi et al. (2025)	Immunological Insights into the PI3K-Akt Pathway in Osteoporosis and Periodontitis: A Proteomic and Metabolomic Approach	Does not address MRONJ
67	Yu et al. (2024)	OSTEO18, a novel urinary proteomic signature, associated with osteoporosis in heart transplant recipients	Does not address MRONJ
68	Aparicio-Bautista et al. (2024)	Label-free quantitative proteomics in serum reveals candidate biomarkers associated with low bone mineral density in Mexican postmenopausal women	Does not address MRONJ
69	Fretwurst et al. (2022)	Proteomic profiling of human bone from different anatomical sites–A pilot study	Does not address MRONJ
70	Nakai et al. (2024)	Use of data-independent acquisition mass spectrometry to identify an objective serum indicator of the need for osteoporotic therapeutic intervention	Does not address MRONJ
71	Martínez-Aguilar et al. (2019)	Serum Proteomic Analysis Reveals Vitamin D-Binding Protein (VDBP) as a Potential Biomarker for Low Bone Mineral Density in Mexican Postmenopausal Women	Does not address MRONJ
72	W. H. Ho et al. (2012)	Proteomic identification of a novel Hsp90-containing protein–mineral complex which can be induced in cells in response to massive calcium influx	Does not address MRONJ
73	L. Wang et al. (2025)	Differential Proteomics Reveals Kaempferol's Mechanism Against Postmenopausal Osteoporosis	Does not address MRONJ
74	Wang et al. (2025)	Circulating Proteins and Bone Mineral Density: A Proteome-Wide Mendelian Randomization Study	Does not address MRONJ
75	Marx et al. (2023)	Urinary collagen peptides: Source of markers for bone metabolic processes in kidney transplant recipients	Does not address MRONJ
76	Sanchez et al. (2018)	Proteomic analysis of osteoblasts secretome provides new insights in mechanisms underlying osteoarthritis subchondral bone sclerosis	Does not address MRONJ
77	Onono et al. (2010)	A tagging-via-substrate approach to detect the farnesylated proteome using two-dimensional electrophoresis coupled with Western blotting	Does not address MRONJ

78	Song et al. (2024)	Boosting synergism of chemo- and immuno-therapies via switching paclitaxel-induced apoptosis to mevalonate metabolism-triggered ferroptosis by bisphosphonate coordination lipid nanogranules	Does not address MRONJ
79	Panés et al. (2021)	Mechanisms of Non-Response to Adalimumab on Inflammatory Bowel Disease: Peripheral Proteomic and Transcriptomic Profiling from the SERENE-CD and SERENE-UC Studies	Does not address MRONJ
80	Tassone et al. (2009)	Challenging the current approaches to multiple myeloma-related bone disease: from bisphosphonates to target therapy	Does not address MRONJ
81	Hosfield et al. (2004)	Structural basis for bisphosphonate-mediated inhibition of isoprenoid biosynthesis	Does not address MRONJ
82	Karlic et al. (2015)	Inhibition of the mevalonate pathway affects epigenetic regulation in cancer cells	Does not address MRONJ
83	Kane et al. (2021)	The Antifungal and Synergistic Effect of Bisphosphonates in Cryptococcus	Does not address MRONJ
84	Montalvetti et al. (2001)	Bisphosphonates are potent inhibitors of Trypanosoma cruzi farnesyl pyrophosphate synthase	Does not address MRONJ
85	Ling et al. (2007)	The farnesyl-diphosphate/geranylgeranyl-diphosphate synthase of Toxoplasma gondii is a bifunctional enzyme and a molecular target of bisphosphonates	Does not address MRONJ
86	Onay et al. (2023)	Repurposing bisphosphonates for reducing skin inflammation and promoting skin repair	Does not address MRONJ
87	De Lima-Souza et al. (2022)	RISKS AND BENEFITS OF BISPHOSPHONATES: ARE PATIENTS WELL INFORMED?	Does not address MRONJ
88	Bagan et al. (2013)	Bisphosphonates-related osteonecrosis of the jaws: a preliminary study of salivary interleukins	Does not address proteome
89	Sobczak-Jaskow et al. (2023)	A Study of Oral Health Parameters and the Properties and Composition of Saliva in Oncological Patients with and without Medication-Related Osteonecrosis of the Jaw Who Take Bisphosphonates	Does not address proteome

90	Koth et al. (2017)	Interrelationship of clinical, radiographic and haematological features in patients under bisphosphonate therapy	Does not address proteome
91	Jahnke et al. (2010)	Allosteric non-bisphosphonate FPPS inhibitors identified by fragment-based Discovery	Does not address proteome
92	Lu et al. (2025)	Bisphosphonates Trigger Anti-Ageing Effects Across Multiple Cell Types and Protect Against Senescence	No peer review was performe d
93	Marx et al. (2022)	Urinary collagen-derived peptides as sensitive markers for bone resorption and bisphosphonate treatment in kidney transplant patients	No peer review was performe d
94	Shi et al. (2025)	Vascular and Lymphatic Dysregulation via Non-EndoMT Col2a1 Signaling in Bisphosphonate-Related Osteonecrosis of the Jaw	No peer review was performe d
95	Del Real et al. (2022)	Analysis of serum proteome after treatment of osteoporosis with anabolic or antiresorptive drugs	Does not address MRONJ
96	X. Lin et al. (2025)	Proteomic profiling of bone tissue reveals distinct pathways in men and women with osteoporosis	Does not address MRONJ
97	Del Real et al. (2022)	Analysis of serum proteome after treatment of osteoporosis with anabolic or antiresorptive drugs	Does not address MRONJ
98		Proteomic biomarkers associated with low bone mineral density: a systematic review	Review
99	Zhang et al. (2010)	Proteomics in bone research	Review
100	Balachandran et al.	A Mixed Method Systematic Review and Meta-Analysis on Biomarkers for Bisphosphonate-Related Osteonecrosis of the Jaw (Bronj): What the Past Has Seen?	Review
101	Santini et al. (2006)	Mechanisms of disease: preclinical reports of antineoplastic synergistic action of bisphosphonates	Review

102	Galazis et al. (2013)	Proteomic biomarkers of preterm birth risk in women with polycystic ovary syndrome (PCOS): a systematic review and biomarker database integration	Review
103	Maruotti et al. (2012)	Bisphosphonates: effects on osteoblast	Review
104	Laputková et al. (2023)	Medication-Related Osteonecrosis of the Jaw: A Systematic Review and a Bioinformatic Analysis	Review
105	(Park et al. (2021)	Phosphonate and bisphosphonate inhibitors of farnesyl pyrophosphate synthases: A structure-guided perspective	Review
106	Masters et al. (2019)	Evolving concepts in bone infection: redefining "biofilm","acute vs. chronic osteomyelitis","the immune proteome" and "local antibiotic therapy"	Review
107	Wood et al. (2014)	Omic-profiling in breast cancer metastasis to bone: implications for mechanisms, biomarkers and treatment	Review
108	Gronich & Rennert, (2013)	Beyond aspirin—cancer prevention with statins, metformin and bisphosphonates	Review

Table S3. Quality analysis of the studies included in the systematic review using the QUADOMICS tool.

**Item**	**Yes**	**No**	**Unclear**	**Not applied**	**Yes**	**No**	**Unclear**	**Not applied**	**Yes**	**No**	**Unclear**	**Not applied**
	**Thumbigere-Math et al. 2015**				**Lorenzo-Pouso et al. 2022**				**Schwartzová et al. 2024**			
1. Were selection criteria clearly described?	X				X				X			
2. Was the spectrum of patients representative of patients who will receive the test in practice?			X				X				X	
3. Was the type of sample fully described?	X				X				X			
4. Were the procedures and timing of biological sample collection with respect to clinical factors described with enough detail?												

4.1. Clinical and physiological factors	X				X				X			
4.2. Diagnostic and treatment procedures.				X				X				X
5. Were handling and pre-analytical procedures reported in sufficient detail and similar for the whole sample? And, if differences in procedures were reported, was their effect on the results assessed?	X				X				X			
6. Is the time period between the reference standard and the index test short enough to reasonably guarantee that the target condition did not change between the two tests?			X				X				X	
7. Is the reference standard likely to correctly classify the target condition?	X				X				X			
8. Did the whole sample or a random selection of the sample receive verification using a reference standard of diagnosis?	X				X				X			
9. Did patients receive the same reference standard regardless of the result of the index test?	X				X				X			
10. Was the execution of the index test described in sufficient detail to permit replication of the test?	X				X				X			
11. Was the execution of the reference standard described in sufficient detail to permit its replication?	X				X				X			
12. Were the index test results interpreted without knowledge of the results of the reference standard?			X				X				X	
13. Were the reference standard results interpreted without knowledge of the results of the index test?			X				X				X	
14. Were the same clinical data available when test results were interpreted as would be available when the test is used in practice?	X				X				X			

https://doi.org/10.3390/xxxxx

15. Were uninterpretable/intermediate test results reported?				X				X				X
16. Is it likely that the presence of overfitting was avoided?		X			X					X		

https://doi.org/10.3390/xxxxx
